# The Relationship Between Altered Mitochondrial DNA Copy Number And Cancer Risk: A Meta-Analysis

**DOI:** 10.1038/srep10039

**Published:** 2015-05-08

**Authors:** Jia Mi, Geng Tian, Shuang Liu, Xianglin Li, Tianhui Ni, Liwei Zhang, Bin Wang

**Affiliations:** 1 Medicine and Pharmacy Research Center; 2 Department of Clinical Medicine; 3 Basic Medical Department; 4 Yantai Affiliated Hospital; 5 Institute of Molecular Imaging, Binzhou Medical University, Yantai, Shandong, China

## Abstract

Currently, a comprehensive assessment between mitochondrial DNA (mtDNA) content and cancer risk is lacking. We designed this meta-analysis to test the hypothesis that altered mtDNA copy number might influence genetic susceptibility to some specific types of cancer. The processes of literature search, eligibility appraisal and data retrieval were independently completed in duplicate. The mtDNA copy number which was dichotomized or classified into tertiles was compared between cancer cases and controls. Twenty-six articles with 38 study groups were analyzed among 6682 cases and 9923 controls. When dichotomizing mtDNA copy number at the median value, there was an 11% increased cancer risk for carriers of high mtDNA content (P = 0.320). By cancer type, high mtDNA content was associated with an increased risk for lymphoma (OR = 1.76; P = 0.023) but a reduced risk for skeleton cancer (OR = 0.39; P = 0.001). Carriers of the 2^nd^ and 3^rd^ tertiles of mtDNA copy number had an 1.74-fold (P = 0.010) and 2.07-fold (P = 0.021) increased risk of lymphoma, respectively. By contrast, there was correspondingly a 56% (P < 0.001) and 80% (P < 0.001) reduced risk of skeleton cancer. Our findings suggested that elevated mtDNA content was associated with a higher risk for lymphoma, but a lower risk for skeleton cancer.

Mitochondrial DNA (mtDNA) is an extra-chromosomal circular, double-stranded, maternally-inherited DNA; it is 16.5 kb in length and encodes for 37 genes, including 2 rRNAs, 13 mRNAs and 22 tRNAs^1^. Lack of protective histones and deficiency in DNA repair capacity render the mtDNA more vulnerable to mutations, and as a feedback the cell will produce multiple copies of mtDNA molecule to antagonize this damage^2^. Somatic mtDNA mutations are frequently observed in many sites of human cancer^3^,^4^, and it gives a reason to expect that high mtDNA copy number might be a logical biomarker implicated in the onset and evolution of carcinogenesis. For example, elevated mtDNA copy number was pre-diagnostically identified in peripheral white blood cells of healthy subjects who developed B-cell non-Hodgkin lymphomas lately^5^. By contrast, subsequent observations argued against this observation by showing that low mtDNA copy number appeared to be associated with an increased risk of renal cell carcinoma^6^,^7^, leading to the existence of tumor site-specific heterogeneity. Even for the same cancer type, it was not without controversy. A recent study by Hofmann *et al*^8^ reported an opposite claim for renal cell carcinoma against two aforementioned previous studies^9^,^10^. However in medical literature a comprehensive assessment between mtDNA content and cancer risk thus far is lacking. To fill this gap in knowledge, we set up a systematic meta-analysis to test the hypothesis that altered mtDNA copy number might influence genetic susceptibility to some specific types of cancer. In addition, we tried to track potential sources of heterogeneity through subgroup and meta-regression analyses.

## Methods & Materials

### Literature search

Potentially eligible articles were obtained through searching the MEDLINE database (http://www.ncbi.nlm.nih.gov/pubmed) by two authors (Jia Mi and Geng Tian). The last update was on January 20, 2015. This meta-analysis collected articles that were exclusively published in English medical journals and involved human subjects only. The key subjects used in literature search were (‘mitochondrial DNA’ OR ‘mtDNA’) AND (‘copy number’ OR ‘content’) AND (‘cancer’ OR ‘carcinoma’ OR ‘neoplasia’ OR ‘adenoma’ OR ‘neoplasm’ OR ‘myeloma’ OR ‘melanoma’ OR ‘lymphoma’ OR ‘leukaemia’ OR ‘leiomyoma’). We also manually scanned the reference lists of major review articles and relevant original articles to find additional citations of interest. We implemented this meta-analysis of the summarized results of individual studies in accordance with the recommended guidelines in the Preferred Reporting Items for Systematic Reviews and Meta-analyses (PRISMA) statement^11^.

### Eligibility

The eligibility of all retrieved articles was appraised by the same two authors (Jia Mi and Geng Tian). If an article cannot be excluded with certainty from its title and/or abstract, full text and supplementary materials when necessary were read to fully interrogate its eligibility, and all uncertainties over eligibility were solved by a discussion in 100% agreement. In case of the same study group with the same clinical endpoint incorporated in more than one article, the article with the largest sample size took precedence.

### Inclusion criteria

The included articles must meet the following necessary criteria concurrently, that is, all types of cancer except for skin cancer constituted the clinical end points (dependent variables); only retrospective or nested case-control studies were considered; distributions of mtDNA copy number should be dichotomized or categorized into tertiles, quartiles, quintiles or more quantiles in the controls, and provided in both cancer cases and controls.

### Exclusion criteria

As most abstracts did not specifically address the topic of our analysis, they were excluded from our full-text review. Articles that provided only mean numbers of mtDNA copy or examined the association of mtDNA content with cancer severity or progression were excluded. In addition, case reports or series, editorial, reviews and non-English articles were also excluded from this meta-analysis.

### Data retrieval

The relevant data from each qualified article were independently retrieved by two authors (Jia Mi and Geng Tian) according to a self-designed data collection form in Excel, including the first author, year of publication, ancestry of study subjects, cancer type, study design, source of controls, sample size of case and control groups, the distributions of mtDNA copy numbers between the two groups, the examined genes in both mtDNA and nuclear DNA and the assay method, and mean levels of age, male gender, body mass index, smoking and drinking in both groups. If an article provided mtDNA copy number between cancer cases and controls by gender, we retrieved them separately as independent study groups in the final analysis.

### Statistics

The association between mtDNA content and cancer risk was investigated in a random-effects model using DerSimonian and Laird method^12^, and risk estimates were expressed as odds ratio (OR) and 95% confidence interval (95% CI). Heterogeneity arising from pooled individual studies was examined using the *I*^2^ statistic. This statistic is defined as the percentage of the observed between-study variability that is due to heterogeneity rather than chance, and it ranges from 0% to 100%. In this meta-analysis, we specified the *I*^2^ statistic of more than 50% as statistically significant, with higher values suggestive of the existence of heterogeneity.

We took two steps to explore the potential sources of heterogeneity. On one hand, we classified all qualified studies into two or more subgroups of homozygous host characteristics according to ancestry of study subjects (mainly Asian and White), gender (male and female), study design (prospective and retrospective case-control studies), source of controls (population-based controls and hospital-based controls) and cancer type, respectively. Only subgroups involving 2 or more groups were summarized. On the other hand, we constructed a meta-regression model by incorporating some continuous characteristics, such as age, gender (male in percentage), body mass index, smoking and drinking as independent variables.

We depicted the Begg’s funnel plot and computed the Egger regression asymmetry test to assess the probability of publication bias. The Egger’s test can identify the asymmetry of funnel plots by determining whether the intercept deviates significantly from zero in regressing the standardized effect estimates against their precision. In addition, the trim-and-fill method was also employed to estimate the number and outcomes of putatively missing studies stemming from publication bias. Significant publication bias was set at a 10% level of Egger test^13^. The above analyses were completed with the use of STATA software v12.0.

## Results

Through layers of identification and assessment, a total of 26 articles were qualified that examined the association of altered mtDNA copy number with cancer risk^5^,^14^,^15^,^16^,^17^,^18^,^19^,^20^,^21^,^22^,^23^,^24^,^25^,^26^,^27^,^28^,^29^,^30^,^31^,^32^,^33^,^34^,^35^,^36^,^37^,^38^. By gender, we classified these 26 articles into 38 independent study groups involving 6682 cancer cases and 9923 controls. Out of 38 study groups, 8 involved both genders, and 15 involved only males and females, respectively. By ancestry, 18 study groups were Asians, 8 whites, 1 African and 11 mixed populations. By cancer type, digestive cancer was examined in 11 study groups, respiratory cancer in 6 groups, urogenital cancer in 6 groups, head and neck cancer in 5 groups, lymphoma in 5 groups, breast cancer in 3 groups and skeleton cancer in 2 groups. By study design, 21 study groups designed prospectively, and 17 study groups retrospectively. By source of controls, 35 study groups involved controls from populations and 3 from hospitals. Baseline characteristics of all qualified studies are shown in Table 1.

Overall, cancer cases were slightly older than controls (age: 59.50 versus 58.53 years, P = 0.024), and no significant differences were observed in gender and body mass index. Cancer cases were more likely to be smokers (53.61% versus 47.48%, P = 0.005) and drinkers (26.92% versus 21.93%, P = 0.090) than controls. Host characteristics of all study populations are shown in Table 2.

**Figure 1:**
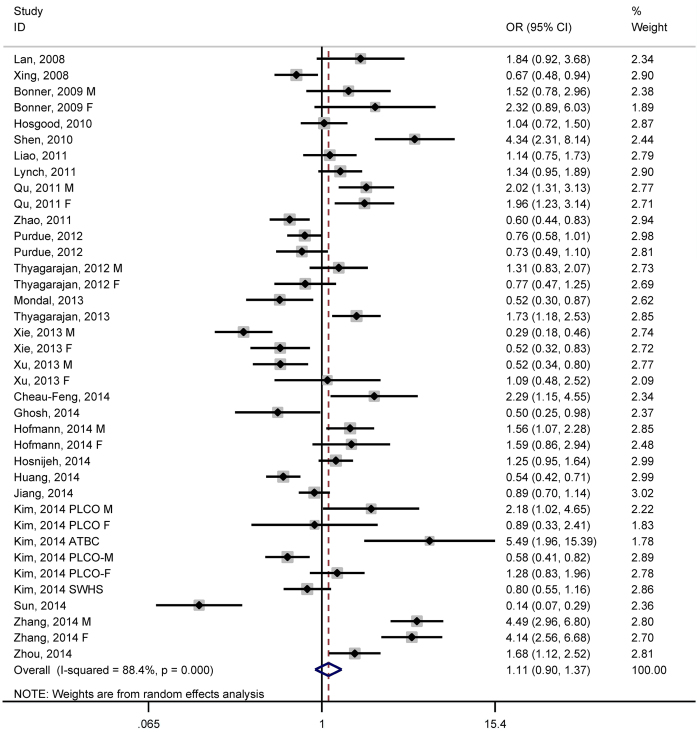
Forest plot of mtDNA and overall cancer risk in median comparison.

**Figure 2:**
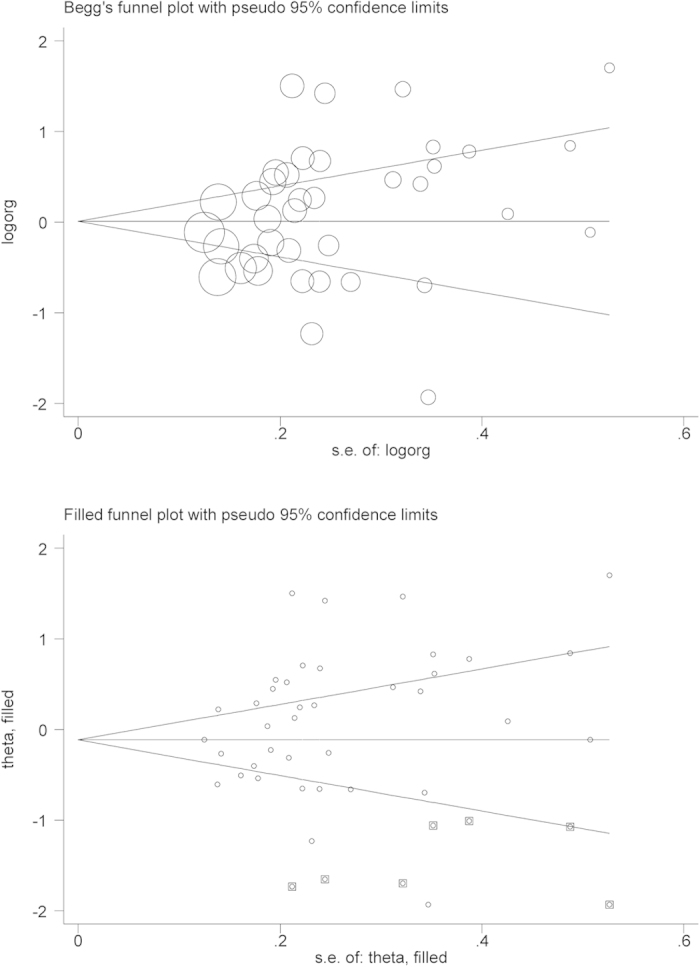
The Begg’s and the Filled funnel plot in median comparison.

When all study groups were pooled together, dichotomizing mtDNA copy number at the median value in the controls identified an 11% increase in overall cancer risk for carriers of high mtDNA content (95% CI: 0.92 to 1.37; P = 0.320) (Fig. 1), with strong evidence of heterogeneity (*I*^2^ = 88.4%) but a low probability of publication bias (P for Egger test = 0.119), although it was estimated to have potentially 7 missing studies stemming from publication bias by the trim-and-fill method (Fig. 2).

A set of subgroup analyses were conducted to account for this evident heterogeneity (Table 3). By ancestry, high mtDNA content appeared to be neutral in populations of White ancestry (OR = 0.97; 95% CI: 0.64 to 1.47; P = 0.894), yet a marginally increased risk was observed in populations of Asian ancestry (OR = 1.29; 95% CI: 0.94 to 1.76; P = 0.116), and no improvement was observed in heterogeneity. By gender, high mtDNA content was consistently overrepresented in cancer cases relative to controls in both genders, especially in males (OR = 1.40; 95% CI: 0.97 to 2.04; P = 0.076), with evident heterogeneity. Grouping studies by study design and source of controls failed to identify any significance. By cancer type, high mtDNA content was associated with an increased risk for lymphoma (OR = 1.76; 95% CI: 1.08 to 2.85; P = 0.023) but a reduced risk for skeleton cancer (OR = 0.39; 95% CI: 0.22 to 0.68; P = 0.001), accompanying moderate heterogeneity (*I*^2^ = 60.3% and 66.7%).

To investigate the possible nonlinear relationship between mtDNA content and cancer risk, we categorized mtDNA copy number into tertiles in the controls and assigned the 1^st^ tertile as the reference (Table 4). Carriers of the 2^nd^ and 3^rd^ tertiles had a linear increase in overall cancer risk, with odds of being 1.31 (95% CI: 0.87 to 1.95; P = 0.192) and 1.45 (95% CI: 0.70 to 2.99; P = 0.313), respectively, without publication bias (P for Egger test = 0.271 and 0.651, respectively). Accordingly as reflected by the trim-and-fill method, there were 2 and 3 missing studies required to achieve symmetry of the Filled funnel plot, respectively (Figures not shown). Still heterogeneity was a disturbing issue for both comparisons (*I*^2^ > 80%).

After grouping studies by ancestry, risk estimates were potentiated for the comparisons of the 2^nd^ and 3^rd^ tertiles with the 1^st^ tertile of mtDNA copy number in populations of both White and Asian ancestries, especially in the former (OR = 1.83 and 2.97; P = 0.081 and 0.045, respectively), with improved heterogeneity. All qualified studies with tertile comparisons enrolled population-based controls. By gender and study design, the association of mtDNA content in tertiles with overall cancer risk was more obvious in males than in females, and in prospective studies than in retrospective studies, while there was no observable significance. By cancer type, carriers of the 2^nd^ and 3^rd^ tertiles of mtDNA copy number had a 1.74-fold (95% CI: 1.14 to 2.65; P = 0.010) and 2.07-fold (95% CI: 1.11 to 3.84; P = 0.021) increased risk of lymphoma, respectively, and heterogeneity was greatly improved (*I*^2^ = 0.0% and 52.6%, respectively). In contrast, there was a 56% (95% CI: 0.30 to 0.63; P < 0.001) and 80% (95% CI: 0.13 to 0.30; P < 0.001) reduced risk of skeleton cancer for the comparisons of the 2^nd^ and 3^rd^ tertiles with the 1^st^ tertile of mtDNA copy number, respectively, and there was no indicative of heterogeneity (both *I*^2^ = 0.0%).

To further study heterogeneity, regression of various study-level covariates was conducted in a multivariable meta-regression model. For both comparisons by median and in tertiles, differences in body mass index explained a marginally significant part of heterogeneity for the association of mtDNA copy number with overall cancer risk (P = 0.065 and 0.081, respectively). In addition, for the comparisons in tertiles, drinking was identified as a potential source of heterogeneity (P = 0.043).

## Discussion

To the best of our knowledge, this is the first systematic meta-analysis covering available English literature to date demonstrating that carriers of high mtDNA content had a higher risk for lymphoma, but a lower risk for skeleton cancer, and this risk prediction might follow a dose-dependent pattern. In spite of a large panel of subgroup and meta-regression analyses, there was no substantial improvement in overall evident heterogeneity for a majority of comparisons in this meta-analysis.

Generally, mitochondria are likened to the energy factories of the cells, and they produce a usable form of energy, adenosinetriphosphate (ATP) through oxidative phosphorylation. Lowered mtDNA copy number can cause a deficiency in oxidative phosphorylation and a resultant enhanced generation of ATP by glycolysis, these changes often implicating cancer development^39^. There is also evidence that mitochondria play a key role in activating apoptosis in mammalian cells^40^ and are the primary target of reactive oxygen species (ROS)^41^. High mtDNA content can be indicative of ROS-mediated oxidative stress, which is thought to be involved in the molecular mechanisms of carcinogenesis^42^,^43^. These observations altogether suggest a biologically plausible role for the changes of mtDNA copy number in the modulation of cancer risk. With the above information in mind, we in the present meta-analysis provided a comprehensive assessment to enrich our understandings of altered mtDNA content in predisposition to overall and specific cancer types.

Of note, our findings revealed a cancer site-specific effect of high mtDNA copy number on the risk of different types of cancer. Our subgroup analysis by cancer type detected a totally opposite outcome between lymphoma and skeleton cancer, with higher mtDNA copy number gradually associating with an increased risk for lymphoma but a reduced risk for skeleton cancer. Understanding tumor heterogeneity may be the next big quest in cancer sciences, which is beyond the capability of the present meta-analysis. However, a phenomenon that cannot be overlooked for lymphoma is that lymphoma is a kind of blood cancer that affects the lymphatic system, and a majority of involved studies quantified mtDNA copy number in peripheral blood lymphocytes, which may better reflect the underlying association between mitochondrial dysfunction and the initiation and progression of lymphoma. This significant association is not surprising, but emphasizes the importance of quantifying mtDNA content in the targeted tissue of each specific cancer, which might be a putative explanation for the neutral associations with the other types of cancer in this meta-analysis. On the other hand, we must have reservations with regard to the association between mtDNA content and skeleton cancer, because this conclusion was only based on two underpowered groups stratified by gender in the study by Xie *et al*^44^. Therefore, the jury must refrain from drawing a firm conclusion until the confirmation from large, well-performed prospective studies with tissue mtDNA content.

### Potential biases and limitations

This meta-analysis is only based on the summary results of published articles, and it is possible some small studies with negative findings are missing. So we cannot exclude the existence of selection bias. Although there was no indication of publication bias according to Egger’s test, the power of identifying significance might be low especially if the total number of studies involved in a meta-analysis is 10 or fewer^45^. The interpretation of our findings might be limited by the strong or moderate evidence of heterogeneity, and this is a common issue with most available meta-analyses in medical literature, leaving further explorations of disturbing heterogeneity open. In addition, the moderate sample size of the current meta-analysis, especially in some subgroups made our findings preliminary and required future confirmation. Furthermore, assay of mtDNA content in peripheral blood lymphocytes may have clouded the true effect of mtDNA copy changes.

### Clinical importance

The utility of mtDNA copy number to indicate the potential for cancer incidence in future screening programs may enable clinical practitioners to better refine individuals at risk for cancer and develop approaches for tailoring antitumor therapy.

In summary, we through a comprehensive meta-analysis demonstrated that elevated mtDNA content was associated with a higher risk for lymphoma, yet a lower risk for skeleton cancer, and the risk prediction followed a dose-dependent pattern. For practical reasons, we hope this study will enrich our understandings of mtDNA content alterations in molecular carcinogenesis. Future investigations to elucidate the specific role of mtDNA in specific cancer are warranted.

## Author Contributions

G.T. and B.W. conceived and designed the experiments; J.M., G.T. and S.L. performed the experiments; G.T. and S.L. analyzed the data; S.L., X.L., T.N. and L.Z. contributed materials/analysis tools; J.M. and B.W. wrote and revised the manuscript. All authors reviewed and approved the manuscript prior to submission.

## Additional Information

**How to cite this article**: Mi, J. *et al*. The Relationship Between Altered Mitochondrial DNA Copy Number And Cancer Risk: A Meta-Analysis. *Sci. Rep.* **5**, 10039; doi: 10.1038/srep10039 (2015).

## Figures and Tables

**Table 1:** Baseline characteristics of all qualified studies in this meta-analysis.

**Author, year**	**Ancestry**	**Control source**	**Match**	**Cancer**		**Gender**	**Study design**	**Sample**	**mtDNA gene**	**Nuclear gene**
				**Detailed type**	**Classification**					
Lan, 2008	White	Population	Yes	Non-Hodgkin lymphoma	Lymphoma	Male	Prospective	PBL	ND1	HGB
Xing, 2008	White	Population	Yes	Renal cell carcinoma	Urogenital	Both	Retrospective	PBL	ND1	HGB
Bonner, 2009 M	Asian	Population	Yes	Lung cancer	Respiratory	Male	Prospective	Sputum	ND1	HGB
Bonner, 2009 F	Asian	Population	Yes	Lung cancer	Respiratory	Female	Prospective	Sputum	ND1	HGB
Hosgood, 2010	White	Population	Yes	Lung cancer	Respiratory	Male	Prospective	PBL	ND1	HGB
Shen, 2010	Mixed	Population	Yes	Breast cancer	Breast	Female	Retrospective	PBL	ND1	HGB
Liao, 2011	Asian	Population	Yes	Gastric cancer	Digestive	Female	Prospective	PBL	ND1	HGB
Lynch, 2011	White	Population	Yes	Pancreatic cancer	Digestive	Male	Prospective	PBL	ND1	HGB
Qu, 2011 Overall	Asian	Population	Yes	Colorectal cancer	Digestive	Both	Retrospective	PBL	ND1	HGB
Qu, 2011 M	Asian	Population	Yes	Colorectal cancer	Digestive	Male	Retrospective	PBL	ND1	HGB
Qu, 2011 F	Asian	Population	Yes	Colorectal cancer	Digestive	Female	Retrospective	PBL	ND1	HGB
Zhao, 2011	Asian	Population	Yes	Hepatocellular carcinoma	Digestive	Both	Retrospective	PBL	ND1	HGB
Purdue, 2012	White	Population	Yes	Renal cell carcinoma	Urogenital	Both	Prospective	PBL	ND1	HGB
Purdue, 2012	African	Population	Yes	Renal cell carcinoma	Urogenital	Both	Prospective	PBL	ND1	HGB
Thyagarajan, 2012 M	Asian	Population	NA	Colorectal cancer	Digestive	Male	Prospective	PBL	ND1	18s
Thyagarajan, 2012 F	Asian	Population	NA	Colorectal cancer	Digestive	Female	Prospective	PBL	ND1	18s
Mondal, 2013	Asian	Hospital	Yes	Oral cancer	Head/neck cancer	Both	Retrospective	PBL	D-loop	GAPDH
Thyagarajan, 2013	Asian	Population	Yes	Breast cancer	Breast	Female	Prospective	PBL	ND1	18s
Xie, 2013 M	Mixed	Population	Yes	Soft tissue sarcoma	Skeleton	Male	Retrospective	PBL	ND1	HGB
Xie, 2013 F	Mixed	Population	Yes	Soft tissue sarcoma	Skeleton	Female	Retrospective	PBL	ND1	HGB
Xu, 2013 M	Mixed	Population	Yes	Esophageal cancer	Digestive	Male	Retrospective	PBL	ND1	HGB
Xu, 2013 F	Mixed	Population	Yes	Esophageal cancer	Digestive	Female	Retrospective	PBL	ND1	HGB
Cheau-Feng, 2014	Asian	Hospital	NA	Head/neck cancer	Head/neck cancer	Male	Retrospective	PBL	tRNA^leu^	18s
Ghosh, 2014	Asian	Hospital	Yes	Nasopharyngeal carcinoma	Head/neck cancer	Both	Retrospective	PBL	D-loop	GAPDH
Hofmann, 2014 M	Mixed	Population	Yes	Renal cell carcinoma	Urogenital	Male	Prospective	PBL	ND1	HGB
Hofmann, 2014 F	Mixed	Population	Yes	Renal cell carcinoma	Urogenital	Female	Prospective	PBL	ND1	HGB
Hosnijeh, 2014	White	Population	Yes	Non-Hodgkin lymphoma	Lymphoma	Both	Prospective	PBL	ND1	HGB
Huang, 2014	Asian	Population	Yes	Colorectal cancer	Digestive	Female	Prospective	PBL	ND1	BRCA1
Jiang, 2014	Asian	Population	Yes	Breast cancer	Breast	Female	Retrospective	PBL	ND1	HGB
Kim, 2014 PLCO M	Mixed	Population	Yes	Non-Hodgkin lymphoma	Lymphoma	Male	Prospective	PBL	ND1	HGB
Kim, 2014 PLCO F	Mixed	Population	Yes	Non-Hodgkin lymphoma	Lymphoma	Female	Prospective	PBL	ND1	HGB
Kim, 2014 ATBC	White	Population	Yes	Non-Hodgkin lymphoma	Lymphoma	Male	Prospective	PBL	ND1	HGB
Kim, 2014 PLCO-M	Mixed	Population	Yes	Lung cancer	Respiratory	Male	Prospective	PBL	ND1	HGB
Kim, 2014 PLCO-F	Mixed	Population	Yes	Lung cancer	Respiratory	Female	Prospective	PBL	ND1	HGB
Kim, 2014 SWHS	Asian	Population	Yes	Lung cancer	Respiratory	Female	Prospective	PBL	ND1	HGB
Sun, 2014	White	Population	Yes	Gastric cancer	Digestive	Both	Retrospective	PBL	ND1	HGB
Zhang, 2014 Overall	Asian	Population	Yes	Glioma	Head/neck cancer	Both	Retrospective	PBL	ND1	HGB
Zhang, 2014 M	Asian	Population	Yes	Glioma	Head/neck cancer	Male	Retrospective	PBL	ND1	HGB
Zhang, 2014 F	Asian	Population	Yes	Glioma	Head/neck cancer	Female	Retrospective	PBL	ND1	HGB
Zhou, 2014	Asian	Population	Yes	Prostate cancer	Urogenital	Male	Retrospective	PBL	ND1	HGB

Abbreviations: M, male; F, female; PBL, peripheral blood lymphocytes.

**Table 2:** Host characteristics of study groups in this meta-analysis.

**Author, year**	**Sample size**		**Age, yrs**		**Male, %**		**BMI, kg/m**^**2**^		**Smoking, %**		**Drinking, %**	
	**Cases**	**Controls**	**Cases**	**Controls**	**Cases**	**Controls**	**Cases**	**Controls**	**Cases**	**Controls**	**Cases**	**Controls**
Lan, 2008	104	104	58.0	57.0	100.0	100.0	25.5	25.4	100.0	100.0	NR	NR
Xing, 2008	260	281	59.2	59.5	66.0	62.0	NR	NR	48.0	55.0	NR	NR
Bonner, 2009 M	73	68	54.9	54.5	100.0	100.0	NR	NR	NR	NR	NR	NR
Bonner, 2009 F	40	39	54.9	54.5	0.0	0.0	NR	NR	NR	NR	NR	NR
Hosgood, 2010	227	227	58.7	58.4	100.0	100.0	25.6	26.3	100.0	100.0	NR	NR
Shen, 2010	103	103	58.0	56.0	0.0	0.0	27.8	26.4	48.5	47.6	72.8	75.8
Liao, 2011	162	299	61.0	61.0	0.0	0.0	NR	NR	5.6	4.4	1.9	2.0
Lynch, 2011	203	656	58.0	58.0	100.0	100.0	26.2	25.7	100.0	100.0	NR	NR
Qu, 2011 Overall			58.4	58.2	52.8	52.8	23.8	23.6	39.4	32.8	13.1	11.9
Qu, 2011 M	169	169	58.4	58.2	100.0	100.0	23.8	23.6	39.4	32.8	13.1	11.9
Qu, 2011 F	151	151	58.4	58.2	0.0	0.0	23.8	23.6	39.4	32.8	13.1	11.9
Zhao, 2011	274	384	50.1	48.7	86.1	84.4	NR	NR	56.2	49.7	35.8	15.1
Purdue, 2012	445	379	NR	NR	58.0	65.0	NR	NR	66.0	61.0	NR	NR
Purdue, 2012	158	224	NR	NR	69.0	49.0	NR	NR	71.0	69.0	NR	NR
Thyagarajan, 2012 M	92	379	66.1	57.6	100.0	100.0	23.0	22.8	42.9	26.1	23.7	19.5
Thyagarajan, 2012 F	76	495	66.1	57.6	0.0	0.0	23.0	22.8	42.9	26.1	23.7	19.5
Mondal, 2013	124	140	58.0	56.0	79.0	72.1	NR	NR	71.7	53.4	NR	NR
Thyagarajan, 2013	183	529	61.1	61.1	0.0	0.0	NR	NR	NR	NR	NR	NR
Xie, 2013 M	174	180	58.2	58.5	100.0	100.0	NR	NR	37.5	35.5	NR	NR
Xie, 2013 F	151	150	58.2	58.5	0.0	0.0	NR	NR	37.5	35.5	NR	NR
Xu, 2013 M	173	173	62.1	60.9	100.0	100.0	NR	NR	68.8	56.0	NR	NR
Xu, 2013 F	45	45	62.1	60.9	0.0	0.0	NR	NR	68.8	56.0	NR	NR
Cheau-Feng, 2014	67	79	56.0	59.6	100.0	100.0	NR	NR	NR	NR	NR	NR
Ghosh, 2014	64	100	NR	NR	76.6	79.0	NR	NR	67.2	26.0	68.8	49.0
Hofmann, 2014 M	164	231	NR	NR	100.0	100.0	NR	NR	60.0	54.7	NR	NR
Hofmann, 2014 F	67	137	NR	NR	0.0	0.0	NR	NR	60.0	54.7	NR	NR
Hosnijeh, 2014	469	469	56.6	56.6	49.3	49.3	26.9	26.6	57.9	56.3	NR	NR
Huang, 2014	444	1423	58.6	55.2	0.0	0.0	24.6	24.4	2.5	3.2	3.2	2.7
Jiang, 2014	506	520	50.0	51.0	0.0	0.0	NR	NR	NR	NR	NR	NR
Kim, 2014 PLCO M	57	185	63.9	63.8	100.0	100.0	27.0	27.3	48.0	51.0	NR	NR
Kim, 2014 PLCO F	38	116	63.9	63.8	0.0	0.0	27.0	27.3	48.0	51.0	NR	NR
Kim, 2014 ATBC	33	97	59.3	57.6	100.0	100.0	26.1	26.4	100.0	100.0	NR	NR
Kim, 2014 PLCO-M	259	267	64.1	63.7	100.0	100.0	26.8	27.4	89.2	54.6	NR	NR
Kim, 2014 PLCO-F	167	169	64.1	63.7	0.0	0.0	26.8	27.4	89.2	54.6	NR	NR
Kim, 2014 SWHS	221	222	59.2	59.2	0.0	0.0	24.6	25.0	7.7	5.0	NR	NR
Sun, 2014	132	125	58.2	55.5	56.1	52.0	NR	NR	41.7	65.1	NR	NR
Zhang, 2014 Overall			NR	NR	58.2	58.2	NR	NR	23.2	20.5	NR	NR
Zhang, 2014 M	241	241	NR	NR	100.0	100.0	NR	NR	23.2	20.5	NR	NR
Zhang, 2014 F	173	173	NR	NR	0.0	0.0	NR	NR	23.2	20.5	NR	NR
Zhou, 2014	193	194	70.3	70.1	100.0	100.0	24.1	23.2	51.8	50.5	NR	NR

Abbreviations: M, male; F, female; BMI, body mass index; NR, not reported.

**Table 3:** Subgroup analyses of mtDNA copy number with cancer risk in median.

**Study groups**	**Studies (n)**	**OR**	**95% CI**	**P**	***I***^**2**^
Ancestry					
Asian	18	1.29	0.94 to 1.76	0.116	89.7%
White	8	0.97	0.64 to 1.47	0.894	88.0%

Gender					
Male	15	1.40	0.97 to 2.04	0.076	89.5%
Female	15	1.27	0.92 to 1.75	0.151	86.8%

Study design					
Prospective	21	1.15	0.94 to 1.39	0.169	75.8%
Retrospective	17	1.02	0.67 to 1.55	0.917	93.2%

Control source					
Population	35	1.14	0.92 to 1.41	0.240	88.8%
Hospital	3	0.83	0.32 to 2.11	0.689	85.1%
Cancer type					

Digestive	11	0.86	0.59 to 1.25	0.432	88.3%
Urogenital	6	1.05	0.74 to 1.49	0.782	79.9%
Lymphoma	5	1.76	1.08 to 2.85	0.023	60.3%
Respiratory	5	1.02	0.73 to 1.43	0.904	68.0%
Head/neck	5	1.63	0.62 to 4.31	0.323	93.9%
Breast	3	1.80	0.81 to 4.01	0.152	92.0%
Skeleton	2	0.39	0.22 to 0.68	0.001	66.7%

Abbreviations: OR, odds ratio; 95% CI, 95% confidence interval.

**Table 4:** Overall and subgroup analyses of mtDNA copy number with cancer risk in tertiles.

**Study groupsStudies (n)**		**High-tertile versus low-tertile**			**Middle-tertile versus low-tertile**				
		**OR**	**95% CI**	**P**	***I***^**2**^	**OR**	**95% CI**	**P**	***I***^**2**^
Overall	11	1.45	0.70 to 2.99	0.313	95.3%	1.31	0.87 to 1.95	0.192	84.5%
Ancestry									
	
Asian	4	1.97	0.66 to 5.92	0.227	97.3%	1.46	0.85 to 2.51	0.171	88.7%
White	2	2.67	1.02 to 8.61	0.045	66.5%	1.83	0.93 to 3.59	0.081	17.6%
Gender									

Male	4	1.34	0.28 to 6.41	0.711	94.4%	1.22	0.46 to 3.20	0.689	85.6%
Female	3	0.88	0.29 to 2.64	0.815	90.4%	1.07	0.57 to 2.01	0.830	36.5%
Study design									

Prospective	5	1.53	0.64 to 3.64	0.338	88.1%	1.39	0.83 to 2.33	0.213	65.2%
Retrospective	6	1.37	0.45 to 4.17	0.578	96.7%	1.22	0.65 to 2.30	0.533	90.0%
Control source									
Population	11	1.45	0.70 to 2.99	0.313	95.3%	1.31	0.87 to 1.95	0.192	84.5%
Cancer type									

Lymphoma	4	2.07	1.11 to 3.84	0.021	52.6%	1.74	1.14 to 2.65	0.010	0.0%
Digestive	3	1.34	0.49 to 3.67	0.571	95.9%	1.26	0.71 to 2.26	0.434	87.8%
Skeleton	2	0.20	0.13 to 0.30	<0.001	0.0%	0.44	0.30 to 0.63	<0.001	0.0%

Abbreviations: OR, odds ratio; 95% CI, 95% confidence interval.
